# Activation of RAS Signalling is Associated with Altered Cell Adhesion in Phaeochromocytoma

**DOI:** 10.3390/ijms21218072

**Published:** 2020-10-29

**Authors:** Hugo M. Rossitti, Ravi Kumar Dutta, Catharina Larsson, Hans K. Ghayee, Peter Söderkvist, Oliver Gimm

**Affiliations:** 1 Department of Biomedical and Clinical Sciences, Linköping University, SE-58183 Linköping, Sweden; hugro684@student.liu.se (H.M.R.); ravi.kumar.dutta@liu.se (R.K.D.); peter.soderkvist@liu.se (P.S.); 2 Department of Oncology-Pathology, Karolinska Institutet, Karolinska University Hospital, Cancer Center Karolinska, SE-17176 Stockholm, Sweden; Catharina.Larsson@ki.se; 3 Department of Clinical Pathology and Cytology, Karolinska University Hospital-Solna, SE-17176 Stockholm, Sweden; 4 Division of Endocrinology, University of Florida and the Malcolm Randall VAMC, Gainesville, FL 32608, USA; Hans.Ghayee@medicine.ufl.edu; 5 Department of Surgery and Department of Biomedical and Clinical Sciences, Linköping University, SE-58183 Linköping, Sweden

**Keywords:** phaeochromocytoma, paraganglioma, hPheo1, NRAS, adhesion, extracellular matrix

## Abstract

Phaeochromocytomas and paragangliomas (PPGLs) are neuroendocrine catecholamine-producing tumours that may progress into inoperable metastatic disease. Treatment options for metastatic disease are limited, indicating a need for functional studies to identify pharmacologically targetable pathophysiological mechanisms, which require biologically relevant experimental models. Recently, a human progenitor phaeochromocytoma cell line named “hPheo1” was established, but its genotype has not been characterised. Performing exome sequencing analysis, we identified a *KIF1B* T827I mutation, and the oncogenic *NRAS* Q61K mutation. While *KIF1B* mutations are recurring somatic events in PPGLs, *NRAS* mutations have hitherto not been detected in PPGLs. Therefore, we aimed to assess its implications for the hPheo1 cell line, and possible relevance for the pathophysiology of PPGLs. We found that transient downregulation of *NRAS* in hPheo1 led to elevated expression of genes associated with cell adhesion, and enhanced adhesion to hPheo1 cells’ extracellular matrix. Analyses of previously published mRNA data from two independent PPGL patient cohorts (212 tissue samples) revealed a subcluster of PPGLs featuring hyperactivated RAS pathway-signalling and under-expression of cell adhesion-related gene expression programs. Thus, we conclude that *NRAS* activity in hPheo1 decreases adhesion to their own extracellular matrix and mirrors a transcriptomic RAS-signalling-related phenomenon in PPGLs.

## 1. Introduction

Phaeochromocytomas and paragangliomas (PPGLs) are neuroendocrine tumours developing from chromaffin cells of the adrenal medulla or embryological remnants of migrating neural crest cells (paraganglia), respectively. In the recent decades, genotyping studies have revealed genotype-phenotype correlations with important implications for evaluating the risk of malignant disease and considering the extent of surgery, i.e., the choice between total and partial adrenalectomy [1,2,3]. However, despite their genetic diversity, most PPGLs appear histologically similar, and while mutational status is a risk factor for development of metastatic disease [3], incurring considerable 10 year-mortality rates of 40−100% [4,5], recent studies suggest that prognosis in terms of overall survival once metastatic disease has developed is unaffected by the mutational status [3,6,7]. Hence, in order to develop new therapeutic strategies, other markers indicating the underlying and targetable pathophysiological mechanisms are needed, which necessitates studies using controlled experimental models.

Transcriptomic analyses have led to the description of two major pathways that drive chromaffin cell tumourigenesis, i.e., pseudohypoxia and receptor tyrosine kinase (RTK) signalling [8,9,10,11,12,13]; and recently Fishbein et al. [14] found that PPGL exhibiting altered WNT-signalling or *MAX* mutations might represent two additional mRNA subtypes. Pseudohypoxia, i.e., activation of hypoxia-inducible factors during normoxic conditions, can be triggered by e.g., mutations in the von Hippel-Lindau gene (*VHL*), hypoxia-inducible factor-2α (HIF2A, gene name *EPAS1*) or succinate dehydrogenase subunits A-D (*SDHA-D*, together referred to as *SDHx*). Pseudohypoxic tumours are usually noradrenergic due to low or lost expression of the enzyme phenylethanolamine- *N*-methyltransferase (PNMT) that converts norepinephrine into epinephrine [15], which may be a sign of dedifferentiation [16]. However, while tumours with mutations in *VHL* are generally benign, those with *SDHB* mutations have an infamously high metastatic potential. On the other hand, dysregulated RTK signalling provides non-physiological mitogenic signals in the RTK/RAS pathway promoting excessive proliferation, and is caused by mutations in e.g., the RTK “rearranged-during-transfection” (*RET*), the rat sarcoma-associated (RAS) protein Harvey-RAS (*HRAS*), or the neurofibromatosis type 1 gene (*NF1*) [12,17]. In general, RTK/RAS-driven tumours are rather benign and well-differentiated in terms of adrenaline production.

hPheo1 is the first (and at the present only) immortalised human progenitor cell line derived from a phaeochromocytoma [18]. It has not been genotypically characterised, but karyotypic analysis revealed loss of chromosome 9p including the *CDKN2A* locus [18], which is negatively altered in 11−24% of PPGL [19,20]. Although hPheo1 cells were derived from a clinically adrenergic tumour, they lack expression of all enzymes required for catecholamine biosynthesis in the reported culture conditions, which could be due to dedifferentiation, or the isolation of an undifferentiated subclone; in both cases the culturing conditions represent an important factor, and in the latter case hPheo1 could provide novel insights not easily acquired with tissue sample data, in which the contributions of rare clones developing new characteristics (e.g., cancer stemness, dedifferentiation, and metastasising capability) are diluted by predominant tumour cell clones, endothelial cells and stromal components. Investigating the human phaeochromocytoma cell line hPheo1 by exome sequencing analysis, we have found that it harbours a mutation in the previously described PPGL susceptibility gene *KIF1B* [21,22], and the *NRAS* Q61K mutation, which is a known oncogenic event in malignant melanoma [23]. Since *NRAS* mutations have not been described in PPGL previously, we aimed to investigate the impact of NRAS in hPheo1, and the relevance of NRAS-related transcriptomic activity concerning the pathophysiology of PPGLs.

## 2. Results

### 2.1. hPheo1 is Heterozygous for NRAS Q61K and Expresses the Mutant Allele

The hPheo1 cell line has been characterized biochemically and karyotypically, and besides a 9p-deletion, including the *CDKN2A* gene locus, no information of other mutations is available [18]. Using whole exome sequencing, we first screened for variants with allele frequencies <0.1% in the population, affecting the following genes that have previously been found mutated or suggested to be susceptibility genes in PPGL [14,24,25]: *ARNT*, *ATRX*, *BAP1*, *BRAF*, *CSDE1*, *EGLN1* (PHD2), *EPAS1* (HIF2A), *FGFR1*, *FH*, *GPR128*, *HRAS*, *KIF1B*, *MAX*, *MYCN*, *MYO5B*, *NF1*, *RET*, *SDHA*, *SDHAF2*, *SDHB*, *SDHC*, *SDHD*, *SLC25A11*, *TCF4*, *TMEM127*, *UBTF*, *VCL*, and *VHL*. After this screening, we only identified a heterozygous *KIF1B* T827I (c.2480C > T, rs121908162) mutation, which has a minor allele frequency of 0.00058 reported in the Genome Aggregation Database (gnomAD). The gene product of *KIF1B* is a postulated tumour suppressor with a role in an apoptotic pathway induced by neurotrophin deprivation [21]. While *KIF1B* was found to be the second most frequently mutated gene in a multicentric Belgian PPGL patient cohort [26], the T827I mutation in exon 24 of the KIF1Bβ-isoform has hitherto only previously been described in a paraganglioma [26] and a neuroblastoma [21]. Assuming that *KIF1B* is a tumour suppressor, the T827I variant’s pathogenicity for hPheo1 appears uncertain since Sanger sequencing of cDNA with an amplicon spanning exons 23−25 showed that mutant and wildtype alleles are both expressed (Supplementary Figure S1), and thereby that the wildtype allele is retained. In addition, the variant is predicted by PolyPhen-2 to be “benign” (score 0.009), by SIFT to “affect protein function” with low confidence (score 0.02), and by MutationTaster to be a polymorphism. Thus, the uncertainty about the function and role of this variant encouraged further analysis of classical cancer genes. This analysis revealed a heterozygous *NRAS* Q61K (c.181C > A, rs1219132549) mutation, which is a well-known oncogenic variant in melanomas and thyroid carcinomas [23] that has not been described in PPGLs previously. The heterozygous nature of the mutation was confirmed with Sanger sequencing of cDNA, showing that both the wild type and mutant alleles are expressed (Supplementary Figure S2). Considering that *HRAS* mutations have been detected in PPGL of the RTK/RAS-driven subtype [12], and that chromaffin cells share embryological origin with melanocytes, which are also susceptible to transformation by *NRAS* Q61 mutations [23], it seems plausible that this *NRAS* gain-of-function mutation might be a previously unknown causative or predisposing factor behind the neoplastic transformation of chromaffin cells.

### 2.2. Downregulating NRAS in hPheo1 Cells Leads to Upregulation of Genes Involved in Cellular Adhesion

**Figure 1:**
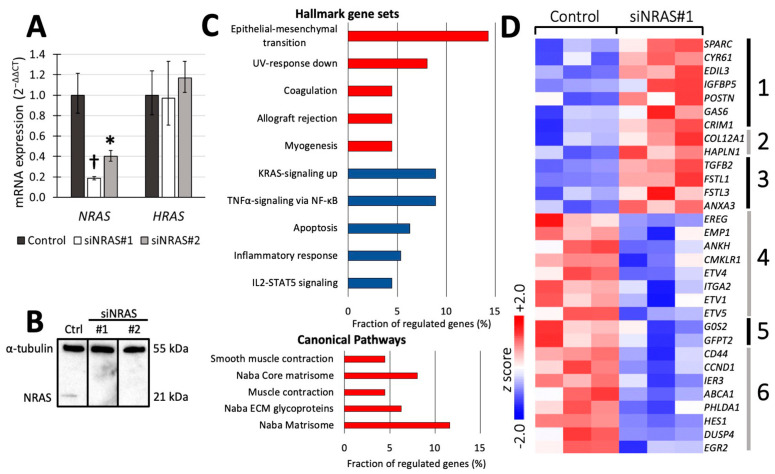
(**A**,**B**) Confirmation of efficient siRNA-mediated knockdown of NRAS expression (siNRAS) at mRNA (**A**) and protein (**B**) level with RT-qPCR and Western blot, respectively, at 72 h post transfection. Two siRNAs were used (siNRAS#1 and siNRAS#2; for sequences, see Materials and Methods). The expression of *HRAS* mRNA (**A**) was unaffected by siNRAS treatment, demonstrating its specificity. In (**A**), mRNA expression in control-transfected (dark-grey bars) and siNRAS-treated (white and light-grey bars) hPheo1 cells is presented as fold change (2^−ΔΔCT^), with results from three independent experiments given as mean ± standard deviation, and *p* values calculated with student’s *T*-test indicated as * (0.001 ≤ *p* < 0.05) or † (*p* < 0.001). In (**B**), representative protein blots from each condition (control (ctrl)); siNRAS#1 and #2 are shown. Alpha-tubulin serves as loading control. (**C**) Top five significant gene sets upregulated (red) and downregulated (blue) in the Hallmark and Canonical Pathways set collections (upper and lower panel, respectively). (**D**) Heatmap displaying the *z* scores of the normalised expression level 2-logs for genes up- or downregulated by siNRAS#1 treatment and belonging to the gene sets Naba matrisome (groups 1−3), Naba core matrisome (groups 1−2), Naba ECM glycoproteins (group 1), Naba matrisome-associated (group 3), Hallmark KRAS-signalling up (groups 4−5), and Hallmark TNFα signalling via NF-κB (groups 5−6). Results from three independent experiments are displayed.

To analyse the effect of *NRAS* in hPheo1 cells, we downregulated its expression using siRNA targeting *NRAS* (henceforth referred to as “siNRAS treatment”), and compared them to cells transfected with a scramble siRNA sequence (“control-transfected” cells). Two siRNAs (siNRAS#1 and #2) were tested, achieving 60−80% reduction in mRNA levels and complete knockdown of NRAS protein expression after 72 h (Figure 1A,B). No change in *HRAS* mRNA expression was observed (Figure 1A), demonstrating the isoform-specificity of the siRNAs. Since siNRAS#1 produced greater effect on the mRNA level, we used this siRNA for microarray and functional studies (detailed below), but all specific gene expression changes were confirmed with both siRNAs.

To investigate the transcriptomic consequences of siNRAS treatment, we used cDNA hybridisation microarrays covering the whole human transcriptome. By comparing siNRAS-treated hPheo1 to control-transfected cells, we detected 119 significantly upregulated transcript cluster IDs and 57 downregulated transcript cluster IDs (Supplementary Tables S1 and S2). Next, we used the Molecular Signature Database (MSigDB) webpage resource [27] to compute significant gene set overlaps among the gene set collections “Hallmark” [28] and “C2: Canonical pathways” [27]. The differentially expressed transcript cluster IDs were identifiable in MSigDB as 112 upregulated and 47 downregulated genes (Supplementary Table S3), which were included in the transcriptomic analyses. When checking for enriched Hallmark gene sets, a significant portion of the upregulated genes were related to epithelial-mesenchymal transition (EMT), whereas a significant portion of the downregulated genes were associated with signalling via KRAS (by extension RAS in general) and TNFα/NF-κB (Figure 1C,D). Furthermore, among the upregulated genes we identified one significantly enriched gene set from the C2: Canonical pathways collection featuring extracellular matrix (ECM) constituents and associated proteins, henceforth referred to as the “matrisome” [29].

**Figure 2:**
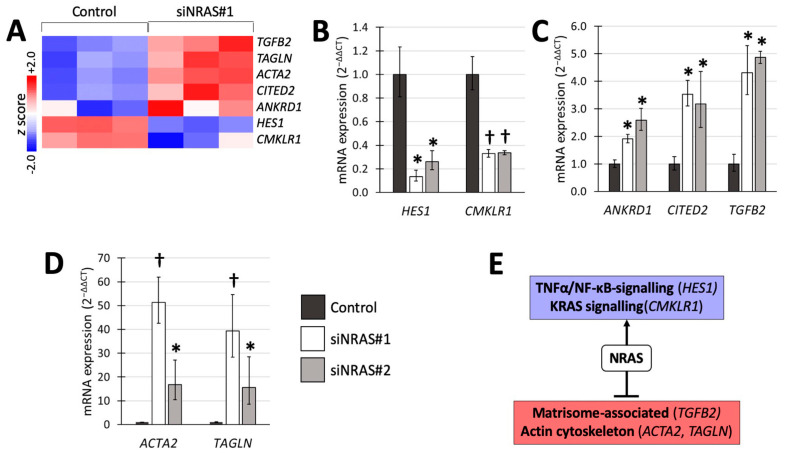
Transcriptomic analysis of siNRAS treatment *versus* control-transfection validated through RT-qPCR using two siRNAs (siNRAS#1 and siNRAS#2). (**A**) Heatmap displaying the *z* scores of the normalised expression level 2-logs for genes that were subsequently validated with RT-qPCR. (**B**) Genes downregulated by siNRAS, and present in the KRAS signalling or TNFα/NF-κB signalling gene sets. (**C**) Upregulation of *TGFB2*, present in the gene set of matrisome-associated proteins, and the genes *ANKRD1* and *CITED2*, by siNRAS. (**D**) Cytoskeleton-related genes upregulated by siNRAS and present in the EMT hallmark gene set. Results from 3 independent siRNA experiments are presented as fold change (2^−ΔΔCT^, mean ± standard deviation), and *p* values calculated with student’s *T*-test indicated as * (0.001 ≤ *p* < 0.05), or † (*p* < 0.001). Bar colours as follows: Control, dark-grey; siNRAS#1, white; siNRAS#2, light-grey. (**E**) Summary indicating the interpretation of the siRNA experiment on transcriptomic data and validated mRNA expression changes: NRAS downregulates matrisomal genes and upregulates TNFα/NF-κB and KRAS-related signalling.

A number of *NRAS* target genes associated with these gene sets, were selected (Figure 2A) for validation with reverse transcriptase quantitative PCR (RT-qPCR) using both siRNAs (Figure 2). We detected 4- to 5-fold upregulation of transforming growth factor-β2 (*TGFB2*) from the matrisomal gene set; 2-fold downregulation of hairy and enhancer of split-1 (*HES1*) from the TNFα/NF-κB-induced gene set; and 2-fold downregulation of chemerin chemokine-like receptor-1 (*CMKLR1*) from the KRAS-induced gene set (Figure 2B,C). In addition, we confirmed upregulation of ankyrin repeat domain-containing protein 1 (*ANKDR1*), the transcription factor *CITED2*, and the EMT-related genes smooth muscle α-actin (α-SMA, also known as *ACTA2*) and transgelin (*TAGLN*) (Figure 2C,D). Figure 2E summarises the transcriptomic findings and validated changes in mRNA expression.

### 2.3. Effects on Cellular Adhesion

**Figure 3:**
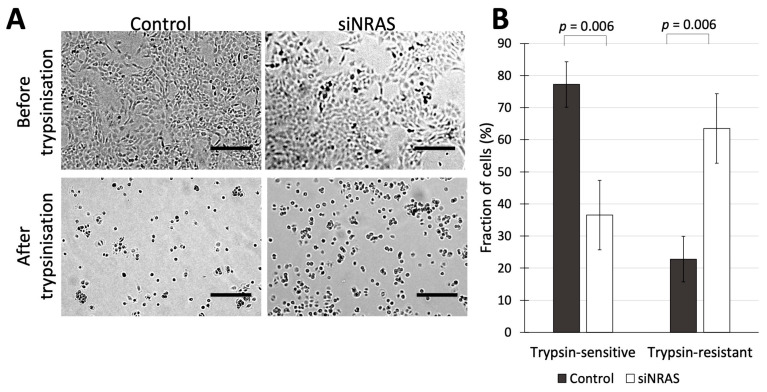
(**A**) hPheo1 cells transfected with control-siRNA or siRNA against *NRAS* (control and siNRAS#1, respectively), before and after trypsinization for 1 min. Scale bars represent 250 μm. (**B**) Cell counts for hPheo1 cells that were detached or remained attached (trypsin-sensitive or trypsin-resistant, respectively) after 1 min of trypsinisation, expressed as percentages of total cell number (mean ± standard deviation, *n* = 3). *p* values calculated with student’s *T*-test.

Since our transcriptomic analysis of siNRAS-treated hPheo1 cells suggested that NRAS activity in hPheo1 cells affects aspects of cell adhesion and ECM-related function, we performed functional experiments to determine those effects using siRNA#1. As an initial approach, we assessed how siNRAS-treatment of hPheo1 affected their trypsin sensitivity, i.e., how sensitive a cell type is to detachment by trypsinisation in vitro, which is a trait that has been shown to distinguish differentially adherent cell types in various contexts, e.g., in cultures of breast cancer, colorectal cancer, and melanoma cells [30,31,32,33]. Differences in trypsin-sensitivity reflects altered expression of trypsin recognition sites on extracellular proteins, which in turn may reflect a large number of alterations at the protein level including ECM remodelling, altered expression of adhesion complexes, and conformational changes of cellular receptors that can alter their affinity for the ECM. We therefore examined how siNRAS treatment affected trypsin sensitivity in hPheo1 cells. First, siNRAS-treated and control-transfected hPheo1 cells were grown until forming a confluent monolayer, and then treated with trypsin for one minute. The number of cells remaining attached to the plate surface (termed “trypsin-resistant” cells), and the number of cells that detached from the plate in the medium (termed “trypsin-sensitive” cells) were counted. We found that *NRAS* downregulation resulted in a 53% decrease in the number of trypsin-sensitive non-adherent cells and a 2.8-fold increase in the number of trypsin-resistant adherent cells (Figure 3).

To determine if the affected cell adhesion factors are intrinsic to the cells or completely extracellular, we performed so-called “spreading assays”, in which freshly plated cells are examined for a “spread” morphology, i.e., the transition from a round unattached cell into a cell with extensions, which indicates the formation of adhesion protein complexes between the cell and the substratum [34]. As a substratum, we prepared plates coated with hPheo1-produced ECM by growing cells to 100% confluence, and then solubilising the cells with NH_4_OH, leaving the ECM intact [35]. With these assays, we aimed to determine (i) how efficiently siNRAS-treated hPheo1 cells adhere to the ECM produced by untreated hPheo1 cells; and (ii) if untreated hPheo1 cells adhere more efficiently to ECMs produced by siNRAS-treated hPheo1 cells.

**Figure 4:**
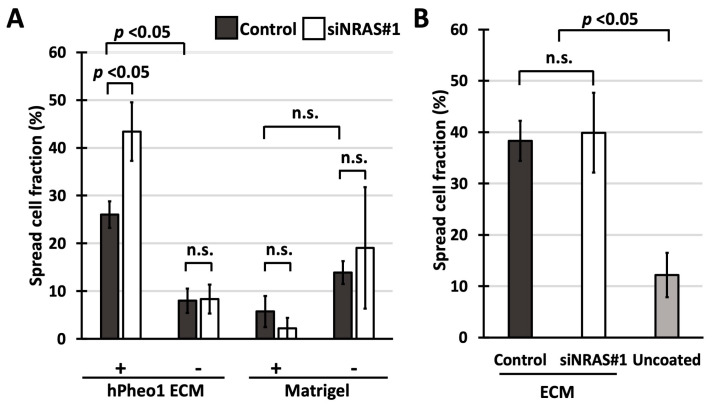
(**A**,**B**) Fraction of cells exhibiting spread morphology presented as percentages (mean ± standard deviation) in spreading assays using cell culture-derived ECMs and Matrigel. (**A**) Spreading of control-transfected and siNRAS#1-treated hPheo1 cells on plates coated with ECMs prepared from untreated hPheo1 cells or Matrigel (+), and uncoated plates (-) after 1 h in serum-free conditions. (**B**) Spreading of untreated hPheo1 cells on ECMs prepared from control-transfected and siNRAS-treated hPheo1 cells (dark-gray and white bars, respectively), and on uncoated wells (light-grey bar) after 1 h in serum-free conditions. All results are from three independent siRNA experiments. If significant one-way ANOVA result, student’s *T*-test *p* values were calculated and corrected ad modum Bonferroni. n.s., no significance.

Adhesive capability was estimated by counting the number of cells that adopted a spread morphology one hour after seeding. As seen in Figure 4A, spreading of siNRAS-treated hPheo1 cells to ECMs produced by untreated hPheo1 cells was 65% more efficient than for control-transfected cells (Bonferroni-corrected *p* = 0.044), a difference not observed for cells plated on uncoated wells. Indeed, both control-transfected and siNRAS-treated cells spread less efficiently on uncoated wells (Bonferroni-corrected *p* = 0.004 for both comparisons), indicating that hPheo1 adheres more efficiently to the decellularised ECM than on plastic, which as a negative control shows that the ECM preparation method is effective. By contrast, no difference was observed when examining the spreading of untreated hPheo1 cells on ECMs from siNRAS-treated hPheo1 (Figure 4B). Further, spreading on Matrigel-coated plates was unaffected by NRAS knockdown, and not significantly different compared to spreading on uncoated plates (one-way ANOVA *p* = 0.056; Figure 4A). Hence, NRAS activity specifically decreases the intrinsic capability of hPheo1 cells to adhere and spread on their own ECM without remodelling it within the observed experimental time frame.

### 2.4. Effects on Proliferation

In the microarray analysis, cyclin D1 (*CCND1*) appeared to be differentially expressed in siNRAS-treated hPheo1 cells and was found among the genes associated with TNFα/NF-κB signalling (Figure 1D, gene group 6). However, this finding could not be validated by RT-qPCR, and the cell proliferation rate was not significantly affected by siNRAS#1 (Supplementary Figure S3A,B).

### 2.5. Molecular Subtype-Specific Gene Expression Patterns

To explore if any NRAS-related transcriptomic patterns observed in hPheo1 cells are present in PPGL tumour samples, we analysed cDNA hybridization microarray data on 26 samples with known driver mutations from a Scandinavian patient cohort [36] (including 5 *EPAS1*-mutated PPGL belonging to a pseudohypoxic subtype; and 11 *NF1*-mutated, 5 *HRAS*-mutated, 3 *RET*-mutated, and 2 *FGFR*-mutated PPGLs belonging to RTK-driven subtypes) and RNA-sequencing data on 186 samples from The Cancer Genome Atlas (TCGA) project [14].

**Figure 5:**
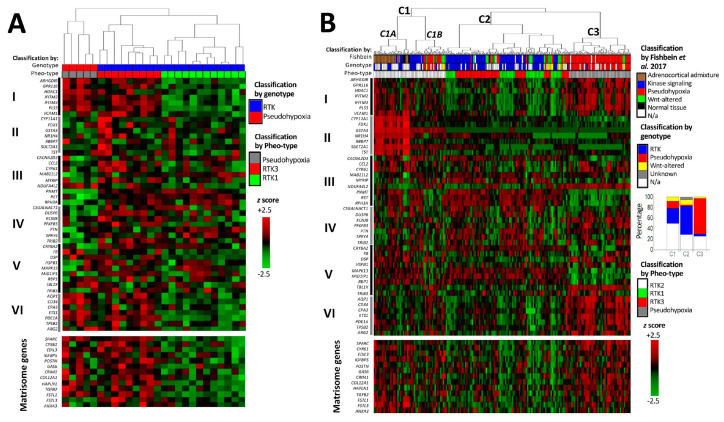
cDNA hybridization microarray data on Scandinavian PPGL with known genotype (**A**) [36] and RNA-sequencing data on The Cancer Genome Atlas cohort (**B**) [14]. The upper heatmap panel displays the expression profile of the Pheo-Type gene set, and the lower panel displays the expression of matrisome-related genes downregulated by NRAS in the hPheo1 cell line. Pheo-Type gene set subdivisions are as described by Flynn et al. [37] and denominated with Roman numerals I–VI (explained in the text). Genotypic subtype indicated as pseudohypoxic (red; *VHL*, *SDHx*, *EPAS1*, *IDH1*, or *EGLN1* mutation), receptor tyrosine kinase (RTK)-driven (blue; *NF1*, *HRAS*, *RET*, *FGFR1*, *TMEM127*, *BRAF*, *NFGR* or *MAX* mutation), WNT-altered (yellow; *CSDE1* or *MAML3* mutation), not applicable (N/a, white), i.e., unknown mutation or known mutation of uncertain mRNA subtype (*SETD2*, *TP53*, *ATRX*). Pheo-Type cluster classification indicated as pseudohypoxic (grey; C3), RTK1 (Green; C2), RTK2 (white; C1A/B) or RTK3 (red; C3). Dendrograms are longitudinally compressed for visualising purposes.

We performed supervised hierarchical clustering based on Pheo-Type, a panel developed by Flynn et al. [37] consisting of 46 genes arranged as six signatures, sets I–VI. In the original work, and as observed in our analysis (Figure 5A,B, upper panels) Pheo-Type identifies the two major mRNA subtypes pseudohypoxic and RTK/RAS-driven PPGLs. This clustering is mainly based on the expression of a chromaffin adrenergic differentiation signature (e.g., *PNMT* and *RET* in gene set III) and a signature featuring endothelial cell markers (e.g., *VCAM1*) related to angiogenesis. While RTK/RAS-driven PPGLs with mutations in *RET*, *HRAS*, and *NF1* are distinguished by overexpression of the adrenergic differentiation signature, pseudohypoxic PPGLs with mutations in *VHL*, *SDHx*, and *EPAS1* are distinguished by low expression of the differentiation signature and concomitant overexpression of the angiogenic signature.

**Figure 6:**
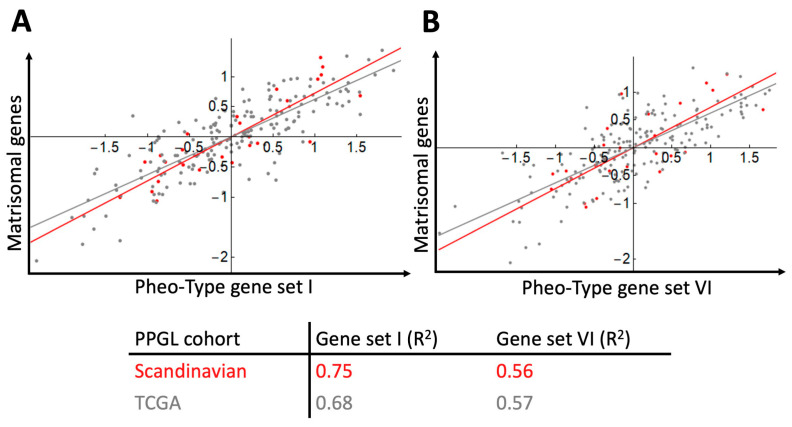
Gene signature scores for PPGL samples (Scandinavian cohort, *n* = 26, red dots and line; TCGA cohort, *n* = 186, grey dots and line) and correlation of the matrisomal gene signature downregulated by NRAS in hPheo1 and the pseudohypoxia-related Pheo-Type gene sets I (**A**) and VI (**B**). Correlation coefficients (R^2^) for each cohort data set are provided.

After categorising the samples according to mRNA subtype, our next step was to examine how the matrisomal signature downregulated by NRAS in hPheo1 correlated with the Pheo-Type signatures. On the expression level heatmaps of the two data sets (Figure 5A,B), we noticed that the angiogenic signature appears to be co-expressed with the matrisomal signature, which we confirmed by correlation analyses yielding correlation coefficients (R^2^) in the range of 0.56–0.75 (Figure 6).

As described by Flynn et al. [37], the RTK/RAS-driven PPGLs in our analyses are further subdivided into three subclusters, denoted as RTK1-3. The RTK2 subcluster highly expresses adrenocortical genes (gene set II), and is considered a cluster of samples with adrenocortical contamination [37], while RTK1 and RTK3 are proper RTK/RAS-driven tumours. Flynn et al. [37] note that RTK3 have higher expression of genes related to inflammatory activity (*CCL2* and *CYR61* in gene set III), and in our analyses they also seem to have higher expression of the angiogenic and NRAS-regulated matrisomal signatures compared to RTK1 tumours. While RTK1 and RTK3 form two separate clusters in our analysis of the Scandinavian cohort (Figure 5A), the RTK1 and RTK3 subclusters in the TCGA cohort were interspersed (Figure 5B(C2)), which can be explained by the TCGA cohort being more genotypically diverse than the cohorts from which the Pheo-Type panel was derived. The TCGA cohort includes PPGLs with altered WNT-signalling clustering with both major clusters (Figure 5B(C2,C3)), and a set of RTK/RAS-driven and pseudohypoxic PPGLs (Figure 5B(C1B)) co-clustering with the adrenocortical admixture samples (Figure 5B(C1A)).

**Figure 7:**
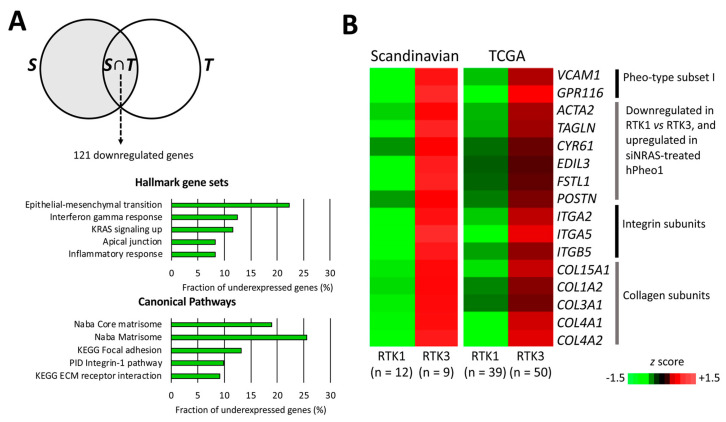
Comparison of RTK1 and RTK3 subclusters. (**A**) Venn diagram (upper panel) illustrating the analysis workflow for patient cohort data. RTK1 tumours were compared to RTK3 tumours in the Scandinavian cohort *S* (grey area). Expression values of significantly under-expressed genes were extracted from the TCGA cohort and the RTK1 and RTK3 clusters were compared (student’s *T*-test *p* < 0.05 with correction ad modum Bonferroni), yielding the intersection of *S* and differentially expressed genes in the TCGA cohort (*T*), i.e., *S*∩*T*, which contained 121 genes significantly downregulated in RTK1 lesions from both cohorts. In the lower panels, the top five significantly under-represented gene sets in RTK1 from the Hallmark (middle panel) and Canonical Pathways set collections (lower panel) are shown. (**B**) Expression of genes downregulated in the RTK1 cluster of both cohorts and either belonging to Pheo-type subset I, or being upregulated by siNRAS treatment of hPheo1 cells, or encoding integrin and collagen subunits; expressed as the mean *z* score for each group.

To better infer the biological differences between RTK1 and RTK3, we compared their transcriptomes in the separate cohorts, and identified a common set of 122 differentially expressed genes (121 downregulated and 1 upregulated; Supplementary Table S4) distinguishing RTK1 (Scandinavian PPGL *n* = 12; TCGA *n* = 39) from RTK3 (Scandinavian PPGL *n* = 9; TCGA *n* = 50). Gene set overlap analysis showed that RTK1 tumours under-expressed genes associated with ECM function, specifically integrin signalling and focal adhesions (Figure 7A). These gene sets include the matrisome- and EMT-related genes *ACTA2*, *CYR61*, *EDIL3*, *FSTL1*, *POSTN*, and *TAGLN* that were also significantly downregulated by NRAS in hPheo1 cells (Figure 1D and Figure 2A; we also note that *CYR61* belongs to gene set III distinguishing the RTK3 subcluster, validating our analysis); *VCAM1* and *GPR116* in Pheo-Type gene set I; integrin subunits α2, α5 and β5; and multiple collagen subunits (Figure 7B). Thus, under-expression of cell adhesion-related genes, including genes downregulated by NRAS in hPheo1 cells, seems to be a feature distinguishing RTK1 from RTK3.

## 3. Discussion

In this study, we report the identification of the missense variant *KIF1B* T827I and the oncogenic mutation *NRAS* Q61K in the hPheo1 cell line. While the *KIF1B* mutation is most likely benign, the *NRAS* mutation is a known oncogenic variant in other neoplastic diseases, and a novel finding for PPGL tumours, for which reason it became the focus of the present study. The most obvious reason to consider the plausibility of *NRAS* gain-of-function being capable of driving chromaffin tumour development is that increased RAS signalling serves as a convergence point for the perturbed signal-transducing processes of RTK/RAS-driven PPGLs [12]. These PPGL typically exhibit an adrenergic secretory profile [15,17,38], to which the clinical phenotype of the patient from whom the hPheo1 cells were derived conforms [18]. Gain-of-function mutations in one of the three mutational hotspots (codons 12, 13 or 61) of RAS proteins are recurrently detected in multiple cancers, in which they provide dysregulated intracellular signals by impairing the self-deactivating GTPase activity common to all small monomeric G-proteins [39]. While the three well-known cancer-associated isoforms *K-*, *N-*, and *HRAS* show >85% amino acid sequence identity indicating functional redundancy, their differential mutation rate in specific tumour types implies cell type-specific roles [23]. The detected *NRAS* Q61K mutation in hPheo1 cells represents an intriguing finding since *NRAS* mutations have not been reported previously in PPGL, unlike *HRAS* mutations that are found in 5−10% of PPGL [17,38,40]. However, the NRAS isoform appears functionally important for neural crest-derived tissues, which includes the peripheral nervous system, adrenal medulla, and melanocytes. Melanocytes are known to be susceptible to neoplastic transformation by oncogenic *NRAS* mutants, since melanocytic neoplasias (naevi and malignant melanomas) frequently harbour *NRAS* Q61R or Q61K mutations [23]. In this context, it is also interesting to note that melanocytic naevi with mutations in the NRAS signalling pathway (*NRAS* or *BRAF* mutations) require additional driver mutations to avoid senescence, e.g., in the *CDKN2A* locus [41]. This prerequisite is fulfilled in the hPheo1 cell line harbouring a macrodeletion featuring the *CDKN2A* locus [18], and loss of the *CDKN2A* locus is a recurring phenomenon in PPGLs [19]. Therefore, one may surmise that chromaffin cells and melanocytes are susceptible to neoplastic transformation through similar pathophysiologic pathways. Moreover, in mice, conditional expression of oncogenic *NRAS* in neural crest-derived tissues leads to development of hyperpigmentation and neurofibromas [42], which are typical manifestations of neurofibromatosis type 1, a disease that sometimes presents with phaeochromocytomas and is caused by loss-of-function mutations in the RAS deactivator *NF1* [43], indicating that NRAS mediates at least some of the effects of *NF1* loss-of-function that cause neurofibromatosis type 1. Somatic mutations in *NF1* are also the most frequent genetic alteration found in sporadic PPGLs [8,44].

We show that downregulation of *NRAS* in hPheo1 cells leads to increased expression of matrisomal and EMT-related genes, and decreased expression of transcriptional targets related to TNFα via NF-κB, which means that NRAS downregulates a matrisomal gene set and upregulates targets of TNFα and NF-κB signalling. All these transcriptomic changes are known to affect the interactions between cells and their microenvironment. Such interactions can be categorised as cell-to-cell and cell-to-ECM interactions, which are the consequences of cellular adhesion mediated by specific adhesion proteins determining the malignant potential at the cellular level and resulting in invasive tumour growth or metastatic disease [45,46]. In malignant melanoma, oncogenic *NRAS* has been shown to affect growth pattern, motility and ECM degradation [47]; and in malignant PPGLs, altered cellular adhesion and ECM remodelling leading to increased tumour cell migration or invasion have been observed, attributed either directly due to malignancy-prone *SDHB* mutations [48,49,50] or accumulation of somatic mutations [24]. Interactions between PPGL tumour cells and cancer-associated fibroblasts have been described in vitro [49] but require further characterisation. A role for NF-κB in chromaffin neoplastic disease progression has been suggested in a study showing that inhibition of NF-κB with triptolide decreases the metastasising capability of phaeochromocytoma cells in a murine model of malignant PPGL dissemination in vivo [51]. Moreover, TNFα signalling combined with RAS hyperactivity may promote tumorigenesis-enabling inflammation in a positive feedback loop that favours cell survival over apoptosis, which has been described in colorectal carcinoma [52] and breast cancer [53].

Although a significant portion of the genes downregulated by NRAS in hPheo1 cells were associated with an “EMT” signature, transcriptomic signatures always require contextualisation. EMT means that epithelial cells change their repertoire of adhesion molecules from epithelial to mesenchymal types [46], and trigger remodelling of the local ECM [54]. Unlike cell types of ecto- and endodermal lineages, for which EMT signatures apply as markers of increased metastasising potential, chromaffin cells are of neuroectodermal origin and undergo a normophysiological EMT during separation from the ectoderm and neural tube before migration into their final anatomical positions [55]. Intriguingly, the proteomic profile of PPGLs most closely resembles that of sarcomas, melanomas, and primary brain tumours by displaying higher expression of so-called “EMT signatures” [56], demonstrating the embryological relatedness of neuroectodermal derivatives and the adoption of mesenchymal-like traits during delamination and migration of neural crest-derived cells. Therefore, we do not infer that NRAS downregulating an “EMT” signature increases the malignant potential of hPheo1, but rather implies perturbed cellular adhesion mechanisms.

Our finding that siNRAS-treated hPheo1 cells are more resistant to detachment through trypsinisation (Figure 3) indicated a change in the expression of trypsin-sensitive extracellular proteins, which are either cellular receptors or ECM components. The in vitro-trait of trypsin-sensitivity has been used to distinguish cell types with different adhesion characteristics, e.g., subpopulations in breast cancer cell cultures, melanocyte cultures and colorectal cancer cell cultures [30,31,32,33]. However, it must be noted that in any context, differences in trypsin sensitivity only indicates altered expression (or exposure) of trypsin target sites on extracellular protein domains in the cell culture, and does not quantify “adhesion” to any substratum. Nevertheless, this finding prompted further investigations into cellular adhesion.

Regarding the effects of NRAS on hPheo1 cells’ adhesion properties, we report two in vitro observations. First, we found that siNRAS-treated hPheo1 cells’ adhesion onto their native ECM is enhanced (Figure 4A). Notably, no difference could be observed when spreading untreated hPheo1 cells on ECMs produced by siNRAS-treated hPheo1 (Figure 4B). Hence, the altered adhesive properties appear intrinsic to the cells, which could be explained by NRAS-driven downregulation of actin cytoskeleton constituents ACTA2 and TAGLN (Figure 2D) influencing the conformations and hence ECM-binding affinities of cellular surface receptors, and downregulation of integrin subunit α2 (ITGA2, Figure 1D) changing the repertoire of ECM-binding integrins.

Second, we report that siNRAS- and control-treated hPheo1 cells adhered with similar efficiency to Matrigel-coated and uncoated plates (Figure 4A). As an informative contrast to our findings, murine chromaffin cells transformed through knockout of the tumour suppressors SDHB [50] or SLC25A11 [25] showed improved spreading on Matrigel-coated and uncoated plates while exhibiting increased malignant potential as expected for pseudohypoxic PPGL with mitochondrial dysfunction. Thus, our findings together indicate that NRAS and by extension RTK-signalling alters cell adhesion differently compared to pseudohypoxic pathways.

The cell-adhesive substrate requires careful consideration when interpreting observations on cellular adhesion. In terms of the differential adhesion hypothesis [45], increased adhesion to Matrigel indicates a higher tendency for tumour cell dissemination in an epithelial basement membrane ECM derived from an undifferentiated murine malignancy of possibly yolk sac endodermal origin [57], which in many model systems correlates with stromal invasion. Conversely, impaired adhesion of chromaffin cells to their native microenvironment induced by NRAS would hypothetically bestow an increased tendency for disseminated growth by affecting cellular migration and invasion. However, such cellular behaviours are of higher complexity involving numerous other factors, e.g., chemotactic stimuli, ECM degradation, and chemical and mechanical properties of different ECMs. Therefore, extended in vitro et vivo studies are necessary to understand the possible role of NRAS and other RTK/RAS signals in PPGL tumourigenesis.

Despite the obvious differences between in vitro cell culture and PPGL samples consisting of tumour, vascular, and stromal cells, after analysing two independent data sets based on two different methods of mRNA quantification (cDNA hybridization microarray and RNA sequencing), we report that the matrisomal gene signature downregulated by NRAS in hPheo1 is positively correlated with expression of angiogenic gene signatures containing endothelial cell markers, and that the RTK1 subcluster of RTK/RAS-driven PPGLs is characterised by low expression of both the matrisomal and angiogenic signatures (Figure 5 and Figure 6). This implicates that the cellular adhesion properties of PPGLs could represent interactions between chromaffin and endothelial cells, and that transcriptomic consequences of RTK/RAS signalling downstream of NRAS in hPheo1 are present in PPGLs. We also show that the RTK1 and RTK3 subclusters described by Flynn et al. [37] can be distinguished by their expression of cellular adhesion-related genes, in particular integrins and focal adhesion signalling, which are under-expressed in the RTK1 subcluster (Figure 7) and involved with cell-ECM binding [58]. Thus, our findings demonstrate that the RTK1 subcluster is characterised by decreased cell-ECM binding, which corresponds to our in vitro observations, indicating that downregulation of the NRAS-regulated matrisomal gene signature coincides with decreased adhesion of hPheo1 cells to their intrinsic ECM. However, studies disentangling the contributions of tumour and stromal cells are needed to determine the specific consequences of individual adhesion molecules.

## 4. Conclusions

In summary, we report that the hPheo1 cell line expresses the oncogenic NRAS Q61K variant, and that NRAS activity downregulates a matrisomal gene signature and decreases cell-ECM adhesion. Analysing the mRNA expression profiles of PPGL samples using a gene panel that distinguishes between pseudohypoxic and RTK/RAS-driven PPGLs, we observed that the matrisomal signature, which is downregulated by NRAS activity in hPheo1 cells, positively correlates with angiogenesis-related genes, and is under-expressed in the RTK1 subcluster of RTK/RAS-driven PPGLs. Thus, our findings indicate that hPheo1 cells can be used to experimentally investigate behaviours of undifferentiated intra-tumoral subclones with decreased cell-ECM adhesion that are relevant to PPGLs; and further implicate that cellular adhesion might represent a biologically interesting basis for further characterising PPGLs in terms of tumour-stroma interactions, cell dissemination, and growth patterns. These aspects of cellular behaviour may reflect clinically relevant parameters like invasive growth and metastatic formation, whose integration with other cellular functions of known importance to PPGL pathophysiology (e.g., mitogenic signalling, metabolism, and DNA methylation) presents an intriguing subject for future studies.

## 5. Materials and Methods

### 5.1. Cell Culture

hPheo1 cells (kindly provided by Professor Jerry W. Shay (Southwestern Medical Center, University of Texas, Dallas, TX, USA) were maintained in RPMI-1640 medium supplemented with 5% foetal bovine serum, 2 mM L-glutamine, 100 units/mL penicillin and 100 μg/mL streptomycin (all cell culturing reagents purchased from Gibco^TM^, Thermo Fisher Scientific, Waltham, MA, USA) and incubated at 37 °C (5% CO_2_). Cells were passaged once per week and counted with a TC10^TM^ Automated Cell Counter after staining with Trypan Blue Dye 0.40% (Bio-Rad, Hercules, CA, USA). Separately thawed clones were used for microarray (passages 25−28), functional studies and RT-qPCR validation (passages 3−15), respectively.

### 5.2. DNA Extraction, Exome and Sanger Sequencing

hPheo1 DNA was extracted with AllPrep DNA/RNA Mini Kit (Qiagen, Hilden, Germany) and quality-checked with a Fragment Analyzer (Advanced Analytical Technologies Inc., Ames, IA, USA). DNA library preparation was performed with SureSelect^XT^ Clinical Research Exome (Agilent Technologies, Santa Clara, CA, USA), and pair-end sequencing (2 × 75 bp) with an Illumina NextSeq 500 instrument (high output mode) according to manufacturer’s instructions (Agilent Technologies). Raw data files were converted into Fastq file format using bcl2Fastq (V2.19 Illumina). Sequencing reads were mapped to human hg19 using Burrows-Wheeler-Aligner (BWA/0.7.15). PCR duplicate removal and calibration of reads were performed using Picard (Picard/2.0.1), variants called using Genome Analysis Toolkit (GATK/3.8-0, HaplotypeCaller) and annotated with ANNOVAR (annovar/2018.04.16). Variants and reported allele fractions were visualised with Integrative Genome Viewer (IGV). Identified mutations were confirmed by Sanger sequencing using MyTaq DNA polymerase (Meridian Bioscience, Cincinnati, OH, USA; see Supplementary Table S5 for primers; Supplementary Table S6 for PCR protocol), ExoSAP-IT (GE Healthcare, Chicago, IL, USA), BigDye Terminator 3.1 for ddNTP-labelling and 3500 Genetic Analyzer for capillary gel electrophoresis (Applied Biosystems^TM^, Thermo Fisher Scientific, Waltham, MA, USA).

### 5.3. RNA Extraction and Quantification

Cells were lysed with Trizol reagent (Invitrogen^TM^, Thermo Fisher Scientific, Waltham, MA, USA), and RNA extracted with the miRNeasy Mini Kit (Qiagen) including the optional step for DNase treatment. RNA was quantified with a NanoDrop1000 instrument. Samples used for microarray analysis had RIN values ≥ 9.9 measured with an Agilent Bioanalyzer.

### 5.4. NRAS Knockdown with siRNA (siNRAS Treatment)

Cells were plated at a density of 0.75−1.0 × 10^4^ cells/cm^2^ and kept in maintenance medium for 24 h before transfection with 1 nM of siRNA 27-mer duplexes (Origene Technologies, Rockville, MD, USA) and 0.15% Dharmafect solution 1 (Dharmacon, Lafayette, CO, USA). Two different siRNAs targeting *NRAS* (siNRAS#1: 5′-AGCUUACUGAUAAACCUAAUAUUCA-3′; siNRAS#2: 5′-CCUGUUAAAUGCUGUAUUUGCUCCA-3′) were used to confirm that changes in gene expression were specific and not off-target effects, and control cells were transfected with scramble control RNA (Catalogue# SR30004, Origene Technologies). After 24 h the transfection medium was replaced with maintenance medium. RNA, protein, and functional studies were performed 72 h after transfection unless specified otherwise.

### 5.5. Western Blot

The cell monolayer was washed with PBS and protein extracted with RIPA buffer (25 mM Tris-HCl pH 7.6, 150 nM NaCl, 1% NP-40, 1% sodium deoxycholate, 0.1% SDS) supplemented with Complete Mini Protease Inhibitor Cocktail (Roche Diagnostics GmbH, Mannheim, Germany). Total protein was quantified with the Pierce^TM^ BCA Protein Assay Kit (Thermo Fisher, Waltham, MA, USA). Duplicate samples (16−20 μg in Laemmli buffer and 1.67% β-mercaptoethanol) were incubated at 98 °C for 5 min; loaded in 4−15% gradient polyacrylamide gels (Mini-PROTEAN^®^ TGX^TM^, Bio-Rad) with TGS running buffer; run on SDS-PAGE at 280 V for 20−25 min; and transferred to PVDF membranes with a Trans-Blot^®^ Turbo^TM^ instrument (Bio-Rad) applying a current of 2.5 A for 3 min. Membranes were blocked in TBS with Tween-20 (TBST) and 5% Blotting-Grade Blocker (Bio-Rad) for 1 h; and incubated in TBST with 5% milk protein at 4 °C overnight (12−14 h) with mouse anti-NRAS antibody diluted 1:100 (sc-31, Santa Cruz Biotechnology Inc., Santa Cruz, CA, USA), and mouse anti-α-tubulin antibody diluted 1:6000 (DM1A, Invitrogen, cat#62204). After three washes in TBST at room temperature (15−20 min each), membranes were incubated at room temperature with anti-mouse antibody (bs-0296G-HRP, Bioss Inc., Woburn, MA, USA) diluted 1:2000 in TBST and 2.5% milk. After three washes with TBST (15−20 min each) and one with TBS (5−10 min), membranes were exposed to Clarity Western ECL Substrate (Bio-Rad), and images developed on a ChemiDoc^TM^ MP* Imaging System for visualisation with ImageLab 4.1.

### 5.6. Microarray Analysis

mRNA expression in siRNA- (siNRAS#1) or control-transfected hPheo1 cells was analysed with the GeneChip HuGene ST 1.0 array, GeneChip^TM^ WT PLUS Reagent Kit, Fluidics Station 450/250, GeneChip^®^ Hybridization Wash and Stain Kit, and GeneChip^®^ Scanner 3000 (Affymetrix, Santa Clara, CA, USA). CEL files were quality-checked with Expression Console (Thermo Fisher; analysis settings: RMA, “gene level”), and CHP files analysed with Transcription Analysis Console (Thermo Fisher). Genes with *p* values < 0.05 (ANOVA), FDR-corrected *p* values < 0.25, and fold change < −1.5 or >1.5 were considered significantly regulated genes. Gene set overlaps were computed using the Molecular Signatures database webpage (MSigDB v6.2) [27] for the “Hallmark” [28] and “C2: Canonical pathways” [27] gene sets, with *p* values calculated from the hypergeometric distribution and corrected for multiple testing with the Benjamini and Hochberg procedure. Heatmaps displaying the expression of genes of interest were generated with Wolfram Mathematica version 11.1 (Wolfram Research, Champaign, IL, USA).

### 5.7. Real-Time Reverse Transcriptase Quantitative PCR

mRNA quantitation by real-time reverse transcriptase quantitative PCR (RT-qPCR) was performed with the 2^−ΔΔCT^ method [59] for *ACTA2*, *ANKRD1*, *CCND1*, *CITED2*, *CMKLR1*, *HES1*, *HRAS*, *NRAS*, *TAGLN*, and *TGFB2*. Primers were designed using Primer Express software version 3.0 (Applied Biosystems), aiming for amplicon lengths of 50−75 bp extending across exon-exon boundaries (see Supplementary Table S5 for primer sequences). Sequence specificity was checked with BLAST. Primers were validated by comparing their amplification efficiencies to primers for reference genes (β-glucuronidase (*GUSB*) and hypoxanthine phosphoribosyl transferase-1 (*HPRT1*)) as recommended in the Real-time PCR Handbook (Life Technologies, Thermo Fisher Scientific; see Supplementary Table S5). RNA was converted to cDNA using Maxima^®^ First Strand cDNA Synthesis Kit (Thermo Scientific). RT-qPCR reactions were run on a 7500 Fast Real-Time PCR System with *Power* SYBR^®^ Green PCR Master Mix (Applied Biosystems), and 450 nM of each primer (see Supplementary Table S6 for cycling protocol); followed immediately by melt curve or agarose gel electrophoresis analysis to ensure amplicon specificity. Threshold cycle (CT) values were acquired with 7500 Software v2.3 (Applied Biosystems). Each sample was run in technical duplicates.

### 5.8. Patient Cohort Analyses

Previously published cDNA hybridization microarray data on 26 tumour samples with known genotype from a Scandinavian PPGL patient cohort [36], and RNA-sequencing data on 186 samples from The Cancer Genome Atlas (TCGA) project [14] were analysed. Scandinavian PPGL data (GeneChip HuGene ST 1.0 array, Affymetrix) were processed as described above for hPheo1 microarrays, whereas TCGA FPKM-UQ data were log2-transformed (with entries of “0” reads set to 1, giving Log_2_(1) = 0), prior to mRNA subtype classification based on their expression of the Pheo-Type gene set [37] by supervised hierarchical clustering with Euclidean distance function and Ward linkage in Wolfram Mathematica version 11.1. After clustering, enriched gene sets affected by siRNA targeting *NRAS* (siNRAS) of hPheo1 cells were scrutinised for molecular subtype-related expression patterns. To acquire a set of genes differentially expressed in both cohorts between the RTK1 and RTK3 subtypes, the Scandinavian PPGLs were analysed as described for hPheo1 cells, but including only transcripts with fold changes < −2 or >2; and the expression values of those genes were extracted from the TCGA data, log2-transformed and tested for significance using student’s T-test with Bonferroni correction. Genes significantly differentially expressed in both comparisons were checked for gene set overlaps in MSigDB as described above. Heatmaps illustrate gene expression as *z* scores, i.e., the number of standard deviations a value deviates from the mean of the whole data set. Gene signature scores were calculated as the mean of the *z* scores of the genes in each signature, and linear regression performed in Wolfram Mathematica version 11.1.

### 5.9. Proliferation

Transfected hPheo1 cells were split 48 h after transfection and re-plated in 6-well plates (4−5 × 10^4^ cells/9 cm^2^). At 24 h-intervals corresponding to 72, 96 and 120 h after transfection, cells were trypsinised for 5 min, stained with trypan blue and counted.

### 5.10. Functional Adhesion Studies

Trypsin sensitivity was assessed on 9 cm^2^ wells by treating hPheo1 cells with 0.05% trypsin and 0.18 mM EDTA (Gibco^TM^, Thermo Fisher Scientific) diluted in PBS for 1 min at 37 °C. Detached trypsin-sensitive cells were stained with Trypan blue and counted with a TC-10 cell counter (Bio-Rad), whereas the adherent trypsin-resistant cells were provided medium to inactivate residual trypsin. After 30 min at 37 °C the medium was replaced to remove nonadherent unviable cells, and cells were photographed with an inverted phase contrast light microscope with a 4X objective. Images were visualised with ImageJ software and cells ≥ 10 μm in diameter (area ≥ 78 μm^2^) were counted using the “Analyse Particles” option after thresholding to only display the cells. Means were calculated from three fields per well and multiplied by 100 (each field corresponding to 1% of the whole well) to estimate the average cell number per well.

Spreading assays were performed on polystyrene wells coated with cell culture-derived extracellular matrices (ECMs) prepared as described by Hellewell et al. [35], or with a 0.5 mm layer of Matrigel (9 mg/mL; Corning^®^, Corning, NY, USA). Cells were plated at a density of 3−4 × 10^4^ cells/9 cm^2^ on ECM-coated or uncoated wells in RPMI-1640 medium without supplements to avoid confounding by unspecific adsorption of seral proteins. One hour after plating, cells were washed with PBS, provided with new RPMI-1640 medium, and photographed with an inverted phase-contrast light microscope (4× objective). The fraction of cells exhibiting spread morphology, i.e., the adoption of a non-rounded nuclear shape and/or development of cytoplasmic flat extensions around the nucleus (as analysed by Loriot et al. [50] and Buffet et al. [25] in studies on murine phaeochromocytoma cells), was assessed in three representative images per well.

### 5.11. Statistical Analyses

The means of two groups (ΔCT values and cell counts) were compared using two-tailed student’s *T*-test. Multiple groups were compared using one-way ANOVA followed by two-tailed student’s *T*-test with correction ad modum Bonferroni. Results with *p* values < 0.05 were considered statistically significant. All cell culture analyses are based on three independent experiments.

## Supplementary Materials

Supplementary Materials can be found at https://www.mdpi.com/1422-0067/21/21/8072/s1, Figure S1: Confirmation of *KIF1B* T827I mutation in hPheo1 cell line through Sanger sequencing; Figure S2: Confirmation of *NRAS* Q61K mutation in hPheo1 cell line through Sanger sequencing; Figure S3: CCND1 gene expression and hPheo1 proliferation; Table S1: List of transcript cluster IDs significantly upregulated in hPheo1 by siNRAS treatment; Table S2: List of transcript cluster IDs significantly downregulated in hPheo1 by siNRAS treatment; Table S3: Genes up- and downregulated by siNRAS-treatment of hPheo1; Table S4: Differentially expressed genes in RTK1 and RTK3 PPGL subclusters; Table S5: Primer pairs; Table S6: PCR protocols.
