# Supplementary material for: Activation of RAS Signalling is Associated with Altered Cell Adhesion in Phaeochromocytoma
Source: Int J Mol Sci. 2020 Oct 29;21(21):8072. doi: 10.3390/ijms21218072 (PMC7663737; doi:10.3390/ijms21218072)
Supplement: Supplementary file 1 [file ijms-21-08072-s001.pdf]

Supplementary Information for "*Activation of RAS Signalling is Associated with Altered Cell Adhesion in Phaeochromocytoma*" by Hugo M. Rossitti, Ravi Kumar Dutta, Catharina Larsson, Hans K. Ghayee, Peter Söderkvist, and Oliver Gimm.

Supplementary Figures

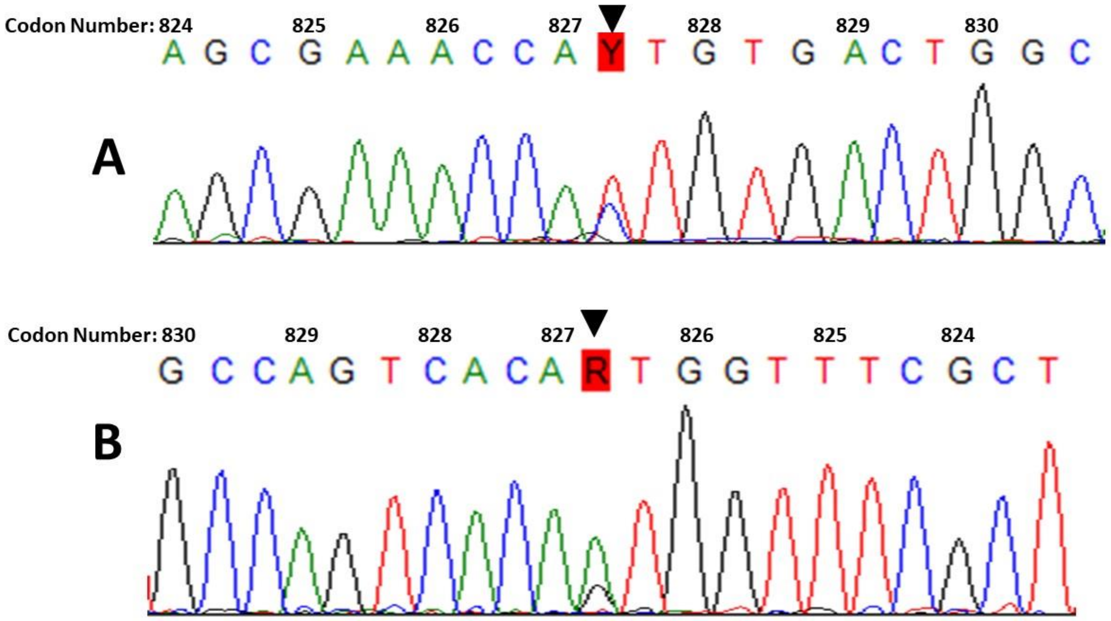

Supplementary Figure S1: Confirmation of KIF1B T827I mutation in hPheo1 cell line through Sanger sequencing. *KIF1B* cDNA sequence trace corresponding to codons 824-830 from hPheo1 cells with forward (coding sequence, ***A***) and reverse primers (complement, ***B***).

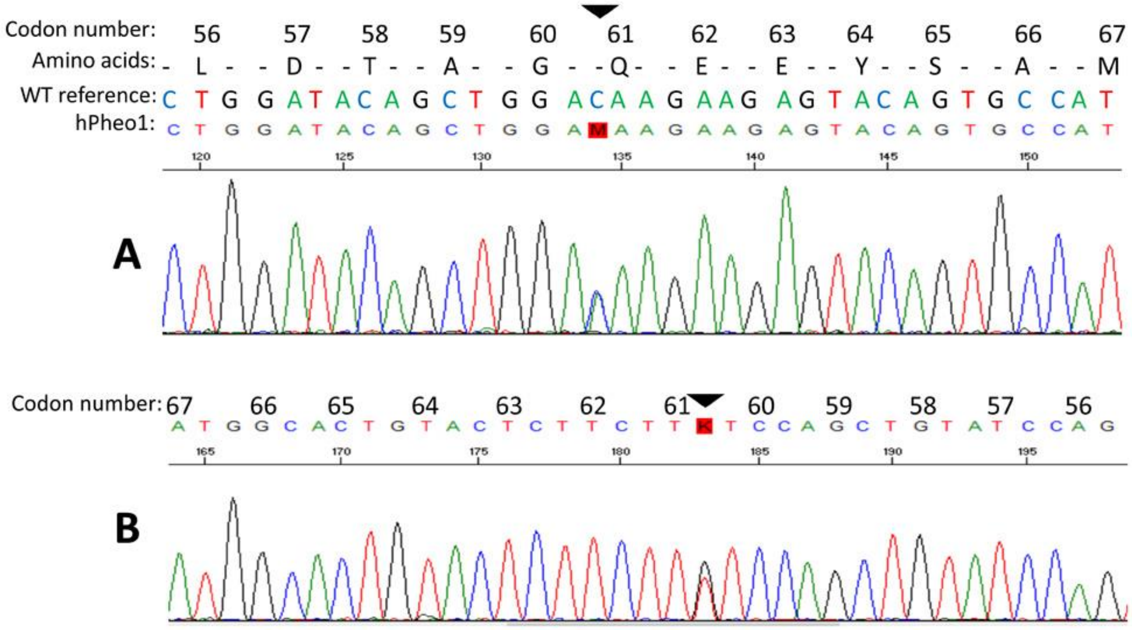

Supplementary Figure S2: Confirmation of NRAS Q61K mutation in hPheo1 cell line through Sanger sequencing. *NRAS* cDNA sequence trace corresponding to codons 56-67 from hPheo1 cells with forward (coding sequence, ***A***) and reverse primers (complement, ***B***).

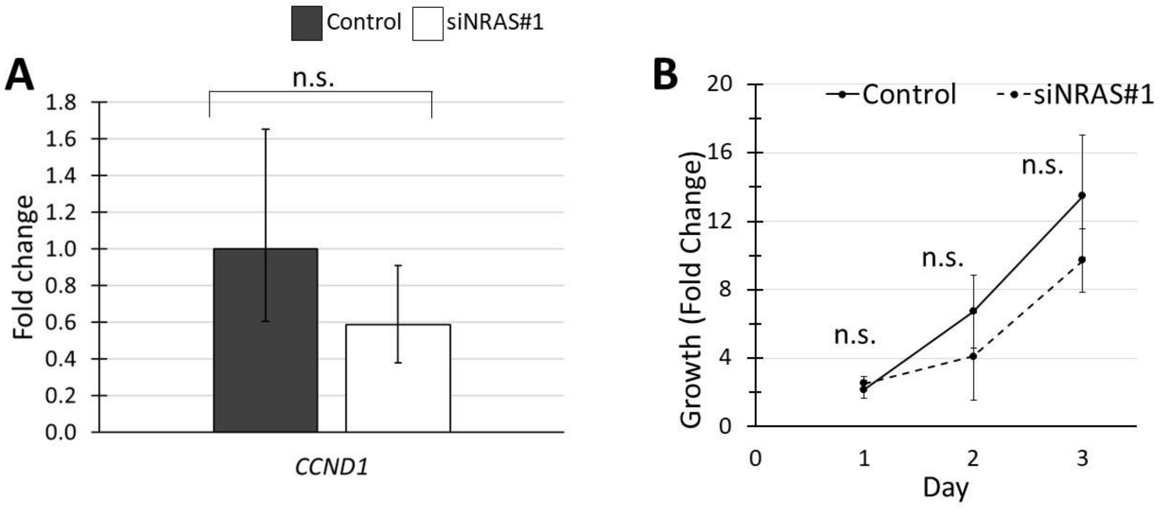

Supplementary Figure S3: CCND1 gene expression and hPheo1 proliferation. A: Expression of *CCND1* mRNA assessed by RT-qPCR and presented as fold change ($2^{-\Delta\Delta CT}$, mean ± standard error of the mean). ***B:*** Cell counts at 1, 2, and 3 days after plating (corresponding to 72, 96 and 120 hours posttransfection, respectively) of control- or siNRAS#1-transfected hPheo1 cells expressed as fold change of the number of cells plated at day 0 (48 hours posttransfection; mean ± standard deviation). All results are from three independent siRNA experiments.

Supplementary Tables

Supplementary Table S1: List of transcript cluster IDs significantly upregulated in hPheo1 by siNRAS treatment (comparison: siNRAS *versus* control-transfected hPheo1; ANOVA $p < 0.05$, FDR < 0.25, fold change < -1.5 or > 1.5).

Transcript Cluster ID	siRNA Average Signal (log2)	Control Average Signal (log2)	Fold Change	ANOVA p-value	FDR p-value	Gene Symbol	Description
8023995	5.6	5.01	1.51	0.001664	0.184761	FSTL3	follistatin-like 3 (secreted glycoprotein)
7964602	6.73	6.13	1.52	0.000493	0.093271	LRIG3	leucine-rich repeats and immunoglobulin-like domains 3
8102831	4.29	3.69	1.52	0.001826	0.196069	MGARP; NDUFC1	mitochondria localized glutamic acid rich protein; NADH dehydrogenase (ubiquinone) 1, subcomplex unknown, 1, 6kDa
8011193	5.11	4.51	1.52	0.000349	0.081128	MIR22HG; MIR22	MIR22 host gene; microRNA 22
8175023	7.43	6.82	1.52	0.000050	0.025635	ZDHHC9	zinc finger, DHHC-type containing 9
8103415	3.55	2.94	1.53	0.000133	0.047180	FAM198B	family with sequence similarity 198, member B
7923453	7.73	7.12	1.53	0.001378	0.165671	KDM5B	lysine (K)-specific demethylase 5B
7915787	6.4	5.78	1.53	0.001383	0.165671	PIK3R3	phosphoinositide-3-kinase, regulatory subunit 3 (gamma)
8094169	4.63	4.02	1.54	0.002276	0.227965	CPEB2	cytoplasmic polyadenylation element binding protein 2
8113504	9.27	8.65	1.54	0.000847	0.124354	NREP	neuronal regeneration related protein
7980616	6.11	5.49	1.54	0.002649	0.248666	PTPN21	protein tyrosine phosphatase, non-receptor type 21
7920057	3.52	2.89	1.54	0.000333	0.081128	TDRKH	tudor and KH domain containing
7923378	10.2	9.57	1.55	0.000210	0.065264	CSRP1	cysteine and glycine-rich protein 1
8026971	8.72	8.09	1.55	0.001293	0.161346	IFI30	interferon, gamma-inducible protein 30

8138091	7.02	6.37	1.56	0.000643	0.110051	DAGLB; KDELR2	diacylglycerol lipase, beta; KDEL (Lys-Asp-Glu-Leu) endoplasmic reticulum protein retention receptor 2
7934997	5.76	5.12	1.56	0.000229	0.066499	PPP1R3C	protein phosphatase 1, regulatory subunit 3C
7912145	7.18	6.54	1.56	0.000472	0.092620	TNFRSF9	tumor necrosis factor receptor superfamily, member 9
8008819	4.84	4.2	1.56	0.000357	0.081128	YPEL2	yippee like 2
8058477	8.98	8.33	1.57	0.000945	0.129746	KLF7	Kruppel-like factor 7 (ubiquitous)
8041206	4.21	3.56	1.57	0.001014	0.137867	LBH	limb bud and heart development
7956856	5.86	5.21	1.57	0.000214	0.065575	MSRB3	methionine sulfoxide reductase B3
8150225	4.68	4.03	1.57	0.000144	0.049037	RAB11FIP1	RAB11 family interacting protein 1 (class I)
7987892	7.42	6.78	1.57	0.000229	0.066499	ZNF106	zinc finger protein 106
7927732	7.23	6.57	1.58	0.000116	0.042733	ARID5B	AT rich interactive domain 5B (MRF1-like)
8111772	7.67	7	1.59	0.000220	0.065907	DAB2; C9	Dab, mitogen-responsive phosphoprotein, homolog 2 (Drosophila); complement component 9
8089544	6.19	5.5	1.61	0.000079	0.034155	CCDC80	coiled-coil domain containing 80
8078461	4.96	4.27	1.61	0.000730	0.115935	FBXL2	F-box and leucine-rich repeat protein 2
8126018	7.04	6.36	1.61	0.000023	0.015824	STK38	serine/threonine kinase 38
8115397	5.67	4.98	1.62	0.000002	0.003708	FAXDC2	fatty acid hydroxylase domain containing 2
8047078	4.92	4.21	1.64	0.000068	0.030938	MFSD6	major facilitator superfamily domain containing 6
7918426	4.25	3.52	1.65	0.000114	0.042432	SLC16A4	solute carrier family 16, member 4
7984079	7.75	7.03	1.65	0.000356	0.081128	TPM1	tropomyosin 1 (alpha)
8175256	5.52	4.79	1.66	0.001428	0.166863	MIR503HG	MIR503 host gene
7956211	10.15	9.41	1.66	0.000002	0.003708	MYL6; MYL6B	myosin light chain 6; myosin light chain 6B

8037537	6.94	6.2	1.67	0.000402	0.085958	ERCC2	excision repair cross-complementation group 2
8156060	6.69	5.96	1.67	0.000094	0.038989	TLE4	transducin-like enhancer of split 4
8178884	6.02	5.27	1.69	0.001314	0.161346	HLA-DMA; HLA-DMB	major histocompatibility complex, class II, DM alpha; major histocompatibility complex, class II, DM beta
8125537	6.16	5.4	1.69	0.002166	0.222582	HLA-DMA; HLA-DMB	major histocompatibility complex, class II, DM alpha; major histocompatibility complex, class II, DM beta
8180086	6.02	5.27	1.69	0.001314	0.161346	HLA-DMA; HLA-DMB	major histocompatibility complex, class II, DM alpha; major histocompatibility complex, class II, DM beta
7952268	9.51	8.76	1.69	0.000011	0.011903	THY1	Thy-1 cell surface antigen
8132710	3.44	2.68	1.7	0.000706	0.114075	C7orf69	chromosome 7 open reading frame 69
8098204	7.42	6.65	1.7	0.000105	0.041776	CPE	carboxypeptidase E
7971294	3.41	2.65	1.7	0.000476	0.092620	FABP3P2	fatty acid binding protein 3, pseudogene 2
7943749	7	6.22	1.71	0.000096	0.039049	LAYN	layilin
7990839	4.9	4.12	1.71	0.000016	0.013804	STARD5	StAR-related lipid transfer domain containing 5
7933821	5.48	4.7	1.72	0.000019	0.014465	RHOBTB1	Rho-related BTB domain containing 1
8090214	7.15	6.36	1.72	1.76E-08	0.000171	SLC12A8	solute carrier family 12, member 8
8127563	7.24	6.44	1.74	0.001390	0.165761	COL12A1	collagen, type XII, alpha 1
8155192	6.02	5.2	1.77	0.000024	0.015824	GLIPR2	GLI pathogenesis-related 2
8108697	4.03	3.2	1.78	0.001850	0.196554	PCDHB5	protocadherin beta 5
8151890	4.07	3.24	1.78	0.000144	0.049037	TP53INP1	tumor protein p53 inducible nuclear protein 1

8155930	5.58	4.74	1.79	0.000795	0.121019	GCNT1	glucosaminyl (N-acetyl) transferase 1, core 2
8127051	9.43	8.57	1.8	0.002729	0.249682	TRAM2	translocation associated membrane protein 2
8117054	8.21	7.35	1.81	0.000014	0.012827	CAP2	CAP, adenylate cyclase-associated protein, 2 (yeast)
8112731	8.76	7.89	1.83	0.000455	0.091250	F2RL2	coagulation factor II (thrombin) receptor-like 2
8097288	5.71	4.83	1.83	0.000877	0.125060	FAT4	FAT atypical cadherin 4
8040473	6.51	5.64	1.84	0.000194	0.063311	RHOB	ras homolog family member B
7933733	5.34	4.46	1.85	0.000335	0.081128	FAM13C	family with sequence similarity 13, member C
7948630	7.77	6.88	1.86	0.000024	0.015824	FADS3	fatty acid desaturase 3
8097829	4.19	3.29	1.86	0.000885	0.125060	FHDC1	FH2 domain containing 1
8077499	3.15	2.26	1.86	0.001596	0.180006	LINC00312; LMCD1	long intergenic non-protein coding RNA 312; LIM and cysteine-rich domains 1
8052654	5.55	4.64	1.89	0.001112	0.147124	PELI1	pellino E3 ubiquitin protein ligase 1
8175263	4.2	3.27	1.9	0.000629	0.108288	PLAC1	placenta specific 1
7921099	6.59	5.66	1.91	0.000184	0.060768	CRABP2	cellular retinoic acid binding protein 2
7925504	4.57	3.62	1.93	0.000597	0.105333	MAP1LC3C	microtubule associated protein 1 light chain 3 gamma
8089835	9.14	8.18	1.94	4.78E-07	0.001420	FSTL1; MIR198	follistatin like 1; microRNA 198
7918323	6.28	5.32	1.94	0.000003	0.004239	SORT1	sortilin 1
8068083	6.32	5.35	1.95	3.05E-07	0.001111	MAP3K7CL	MAP3K7 C-terminal like
8115327	10.79	9.81	1.97	0.000145	0.049037	SPARC	secreted protein, acidic, cysteine-rich (osteonectin)
7970329	6.85	5.86	1.99	0.002423	0.238242	GAS6	growth arrest-specific 6
8155754	6.56	5.57	1.99	0.000920	0.127413	MAMDC2	MAM domain containing 2
8089112	5.55	4.55	2	5.37E-07	0.001420	FILIP1L	filamin A interacting protein 1-like

8117045	5.34	4.34	2.01	0.000241	0.067365	RBM24	RNA binding motif protein 24
7954065	5.99	4.96	2.04	0.000046	0.024237	GPRC5A	G protein-coupled receptor, class C, group 5, member A
7917347	7.65	6.61	2.05	0.000267	0.071720	DDAH1	dimethylarginine dimethylaminohydrolase 1
7905428	5.82	4.79	2.05	1.45E-07	0.000844	TUFT1	tuftelin 1
7973306	7.42	6.37	2.07	0.000469	0.092620	ABHD4	abhydrolase domain containing 4
7977854	6.98	5.93	2.07	0.000033	0.019437	AJUBA	ajuba LIM protein
8141228	5.01	3.97	2.07	0.000434	0.089345	TMEM130	transmembrane protein 130
7942332	3.87	2.8	2.09	0.000042	0.023657	FOLR1	folate receptor 1 (adult)
7962375	4.85	3.77	2.1	0.000019	0.014465	PRICKLE1	prickle homolog 1
8023528	4.25	3.17	2.11	0.000014	0.012790	ALPK2	alpha kinase 2
7942793	4.71	3.61	2.14	0.000418	0.087505	THRSP	thyroid hormone responsive
8098263	7.65	6.53	2.18	0.001067	0.143101	PALLD	palladin, cytoskeletal associated protein
7914557	6.07	4.88	2.28	0.000020	0.014765	SYNC	syncoilin, intermediate filament protein
8123936	5.08	3.86	2.33	0.000031	0.018727	NEDD9	neural precursor cell expressed, developmentally down-regulated 9
7963333	5.97	4.75	2.34	0.000003	0.004787	KRT80	keratin 80, type II
7936050	4.21	2.95	2.41	0.000012	0.012215	CYP17A1	cytochrome P450, family 17, subfamily A, polypeptide 1
8077490	6.84	5.56	2.42	2.69E-07	0.001111	LMCD1	LIM and cysteine-rich domains 1
8116921	5.18	3.86	2.5	0.000011	0.011903	EDN1	endothelin 1
7992828	7.63	6.31	2.5	0.000014	0.012790	IL32	interleukin 32
8089464	4.94	3.61	2.52	0.000131	0.047180	LOC151760	putative uncharacterized protein LOC151760
8155327	8.25	6.89	2.57	0.000020	0.014765	ALDH1B1	aldehyde dehydrogenase 1 family, member B1

8157216	7.91	6.55	2.57	0.000390	0.085542	UGCG	UDP-glucose ceramide glucosyltransferase
8138888	4.69	3.27	2.67	0.000010	0.011903	PDE1C	phosphodiesterase 1C, calmodulin-dependent 70kDa
8041447	8.68	7.22	2.75	0.000135	0.047180	CRIM1	cysteine rich transmembrane BMP regulator 1 (chordin-like)
7967212	5.55	4.07	2.78	0.000031	0.018727	HPD	4-hydroxyphenylpyruvate dioxygenase
8062312	9.71	8.24	2.78	0.000019	0.014465	MYL9	myosin light chain 9
8023497	6.59	5.1	2.81	0.000004	0.005252	ATP8B1	ATPase, aminophospholipid transporter, class I, type 8B, member 1
8129937	7.98	6.48	2.83	0.000002	0.003708	CITED2	Cbp/p300-interacting transactivator, with Glu/Asp rich carboxy-terminal domain, 2
8085138	5.02	3.52	2.83	0.001245	0.157504	OXTR	oxytocin receptor
7950307	7.55	6.05	2.84	0.000023	0.015824	UCP2	uncoupling protein 2 (mitochondrial, proton carrier)
7934979	7.29	5.79	2.85	0.002791	0.249865	ANKRD1	ankyrin repeat domain 1 (cardiac muscle)
7924996	7.48	5.94	2.9	0.000408	0.086002	C1orf198	chromosome 1 open reading frame 198
8136200	8.36	6.82	2.91	8.06E-07	0.001805	CPA4	carboxypeptidase A4
8058857	4.9	3.34	2.93	0.001411	0.165781	IGFBP5	insulin like growth factor binding protein 5
7971077	8.39	6.82	2.97	0.002010	0.210336	POSTN	periostin, osteoblast specific factor
7902687	9.16	7.56	3.04	0.000049	0.025518	CYR61	cysteine-rich, angiogenic inducer, 61
7942503	9.81	8.12	3.21	6.02E-07	0.001458	PPME1	protein phosphatase methylesterase 1
8112980	8.65	6.95	3.25	0.000002	0.003708	EDIL3	EGF-like repeats and discoidin I-like domains 3
8041422	4.33	2.56	3.41	0.000035	0.019965	RASGRP3	RAS guanyl releasing protein 3 (calcium and DAG-regulated)
7923034	3.97	2.15	3.53	0.000009	0.010646	B3GALT2	UDP-Gal:betaGlcNAc beta 1,3-galactosyltransferase 2

7914342	8.02	6.1	3.8	4.12E-09	0.000060	FABP3	fatty acid binding protein 3, muscle and heart
8060897	7.94	6	3.83	0.001656	0.184636	PLCB4	phospholipase C, beta 4
7909789	8.8	6.75	4.14	2.77E-07	0.001111	TGFB2	transforming growth factor beta 2
8095986	7.29	5.04	4.78	0.000030	0.018727	ANXA3	annexin A3
8112971	6.8	3.74	8.35	0.000044	0.023861	HAPLN1	hyaluronan and proteoglycan link protein 1
7944082	8.92	5.09	14.28	5.15E-07	0.001420	TAGLN	transgelin
7934906	10.82	6.86	15.56	1.59E-09	0.000046	ACTA2	actin, alpha 2, smooth muscle, aorta

Supplementary Table S2: List of transcript cluster IDs significantly downregulated in hPheo1 by siNRAS treatment (comparison: siNRAS *versus* control-transfected hPheo1; ANOVA $p < 0.05$, FDR < 0.25, fold change < -1.5 or > 1.5).

**Transcript Cluster ID**	**siRNA Average Signal (log2)**	**Control Average Signal (log2)**	**Fold Change**	**ANOVA p-value**	**FDR p-value**	**Gene Symbol**	**Description**
8117594	7.55	8.91	-2.57	0.000919	0.127413	HIST1H2BM	histone cluster 1, H2bm
8015806	5.76	7.11	-2.56	0.000198	0.064016	ETV4	ets variant 4
8152617	4.59	5.87	-2.44	0.000659	0.111123	HAS2	hyaluronan synthase 2
7918813	6.6	7.87	-2.4	0.000484	0.092620	CSDE1; NRAS	cold shock domain containing E1, RNA binding; neuroblastoma RAS viral (v-ras) oncogene homolog
8105267	5.91	7.14	-2.36	0.000377	0.084364	ITGA2	integrin, alpha 2 (CD49B, alpha 2 subunit of VLA-2 receptor)
7966089	6.35	7.51	-2.24	0.000878	0.125060	CMKLR1	chemerin chemokine-like receptor 1
8149825	5.92	7.08	-2.23	0.000346	0.081128	STC1	stanniocalcin 1
8092578	5.75	6.9	-2.21	0.000333	0.081128	ETV5; DGKG	ets variant 5; diacylglycerol kinase gamma
8138289	5.87	7	-2.18	0.002280	0.227965	ETV1	ets variant 1
7965040	6.91	8.02	-2.16	0.000670	0.112089	PHLDA1	pleckstrin homology-like domain, family A, member 1
8150076	5.21	6.31	-2.15	0.000206	0.065106	DUSP4	dual specificity phosphatase 4
8084880	4.77	5.77	-2	0.000008	0.010200	HES1	hes family bHLH transcription factor 1
7956867	5.39	6.38	-1.99	0.002583	0.245587	HMGA2	high mobility group AT-hook 2
8102200	3.87	4.84	-1.96	0.001303	0.161346	DKK2	dickkopf WNT signaling pathway inhibitor 2
7939341	8.7	9.66	-1.95	0.000066	0.030938	CD44	CD44 molecule (Indian blood group)
7909441	6.95	7.92	-1.95	0.001698	0.187107	G0S2	G0/G1 switch 2
8162216	5.5	6.44	-1.93	0.000017	0.013804	SHC3	SHC (Src homology 2 domain containing) transforming protein 3
8117395	7.75	8.66	-1.88	0.001306	0.161346	HIST1H2BF	histone cluster 1, H2bf
8120967	8.1	9	-1.87	0.002728	0.249682	NT5E	5-nucleotidase, ecto (CD73)
7933872	5.08	5.97	-1.86	0.000750	0.117378	EGR2	early growth response 2

8116418	5.54	6.43	-1.85	0.00115 1	0.15008 5	GFPT2	glutamine-fructose-6-phosphate transaminase 2
7954090	8.06	8.91	-1.81	0.00116 1	0.15008 5	EMP1	epithelial membrane protein 1
8124416	9.13	9.96	-1.77	0.00109 0	0.14479 9	HIST1H3D; HIST1H2AD	histone cluster 1, H3d; histone cluster 1, H2ad
8111101	6.25	7.06	-1.76	0.00078 4	0.12009 9	ANKH	ANKH inorganic pyrophosphate transport regulator
8163618	3.81	4.61	-1.75	3.11E-08	0.00022 6	TNFSF15	tumor necrosis factor (ligand) superfamily, member 15
8117389	2.64	3.43	-1.73	0.00102 5	0.13877 5	HIST1H2BE	histone cluster 1, H2be
7976567	4.56	5.33	-1.71	0.00070 5	0.11407 5	BDKRB1	bradykinin receptor B1
8044499	7.81	8.58	-1.7	0.00054 7	0.10132 9	SLC20A1	solute carrier family 20 (phosphate transporter), member 1
8180308	9.38	10.13	-1.69	0.00241 5	0.23824 2		
8180307	9.36	10.12	-1.69	0.00189 4	0.19894 7		
8095728	4.51	5.25	-1.68	0.00042 4	0.08813 8	EREG	epiregulin
8084891	4.07	4.81	-1.67	0.00176 2	0.19126 0	FAM43A	family with sequence similarity 43, member A
8063923	3.88	4.61	-1.67	0.00250 9	0.24169 8	SLCO4A1	solute carrier organic anion transporter family, member 4A1
8140113	4.74	5.48	-1.67	0.00185 8	0.19655 4	STX1A	syntaxin 1A (brain)
8124524	6.3	7.03	-1.65	0.00278 0	0.24968 2	HIST1H2AK	histone cluster 1, H2ak
8162940	4.3	5.01	-1.64	0.00057 7	0.10359 2	ABCA1	ATP binding cassette subfamily A member 1
8108873	5.09	5.8	-1.64	0.00007 4	0.03310 5	ARHGAP26	Rho GTPase activating protein 26
8178435	9.54	10.25	-1.63	0.00015 5	0.05199 1	IER3	immediate early response 3
8142345	6.24	6.93	-1.62	0.00266 6	0.24880 2	DOCK4	dedicator of cytokinesis 4
8124437	7.98	8.68	-1.62	0.00033 9	0.08112 8	HIST1H3F	histone cluster 1, H3f
8124534	8.7	9.39	-1.62	0.00078 1	0.12009 9	HIST1H4L	histone cluster 1, H4l
8124380	8.62	9.31	-1.61	0.00044 3	0.08949 1	HIST1H1A	histone cluster 1, H1a
7942123	6.27	6.95	-1.6	0.00010 7	0.04177 6	CCND1	cyclin D1
8040238	6.84	7.51	-1.6	0.00004 4	0.02386 1	HPCAL1	hippocalcin-like 1
7949364	5.79	6.44	-1.57	0.00108 3	0.14448 2	CDCA5	cell division cycle associated 5
7898161	6.99	7.64	-1.56	0.00073 6	0.11593 5	EFHD2	EF-hand domain family member D2
7919612	9.75	10.39	-1.56	0.00214 6	0.22216 3	HIST2H3A; HIST2H3D; HIST2H3C; HIST2H3PS2	histone cluster 2, H3a; histone cluster 2, H3d; histone cluster 2, H3c; histone cluster 2, H3, pseudogene 2
7950473	6.73	7.36	-1.55	0.00135 5	0.16422 7	ARRB1	arrestin, beta 1

8179704	9.34	9.97	-1.55	0.000060	0.028764	IER3	immediate early response 3
8124848	9.34	9.97	-1.55	0.000060	0.028764	IER3	immediate early response 3
8180321	8.24	8.87	-1.55	0.000572	0.103347		
8180255	8.24	8.87	-1.55	0.000572	0.103347		
7967117	4.78	5.4	-1.53	0.000089	0.037474	OASL	2-5-oligoadenylate synthetase-like
8017599	4.43	5.04	-1.53	0.000001	0.002300		
8115664	2.82	3.43	-1.53	0.000874	0.125060		
7963545	4.06	4.66	-1.52	0.001354	0.164227	KRT79	keratin 79, type II
8166580	7.23	7.84	-1.52	0.000857	0.124354		

Supplementary Table S3: Genes up- and downregulated by siNRAS-treatment of hPheo1.

Alphabetical list of genes significantly up- and downregulated in hPheo1 by siNRAS-treatment (ANOVA $p < 0.05$, FDR < 0.25, fold change < -1.5 or > 1.5) and mapped in MSigDB, which were included in gene set overlap analyses.

**siNRAS hPheo1**	**Genes**
**Upregulated**	*ABHD4, ACTA2, ALDH1B1, ALPK2, ANKRD1, ANXA3, ARID5B, ATP8B1, B3GALT2, C1orf198, C7orf69, C9, CAP2, CCDC80, CITED2, COL12A1, CPA4, CPE, CPEB2, CRABP2, CRIM1, CSRP1, CYP17A1, CYR61, DAGLB, DDAH1, EDIL3, EDN1, ERCC2, F2RL2, FABP3, FABP3P2, FADS3, FAM13C, FAM198B, FAT4, FBXL2, FHDC1, FILIP1L, FOLR1, FSTL1, FSTL3, GAS6, GCNT1, GLIPR2, GPRC5A, HAPLN1, HLA-DMA, HLA-DMB, HPD, IFI30, IGFBP5, IL32, KDELR2, KDM5B, KLF7, KRT80, LAYN, LBH, LMCD1, LOC151760, LRIG3, MAMDC2, MAP1LC3C, MFSD6, MSRB3, MYL6, MYL6B, MYL9, NDUFC1, NEDD9, OXTR, PALLD, PCDHB5, PDE1C, PELI1, PIK3R3, PLAC1, PLCB4, POSTN, PPME1, PPP1R3C, PRICKLE1, PTPN21, RAB11FIP1, RASGRP3, RBM24, RHOB, RHOBTB1, SLC12A8, SLC16A4, SORT1, SPARC, STARD5, STK38, SYNC, TAGLN, TDRKH, TGFB2, THRSP, THY1, TLE4, TMEM130, TNFRSF9, TP53INP1, TPM1, TRAM2, TUFT1, UCP2, UGCG, YPEL2, ZDHHC9*
**Downregulated**	*ABCA1, ANKH, ARHGAP26, ARRB1, BDKRB1, CCND1, CD44, CDCA5, CMKLR1, DKK2, DOCK4, DUSP4, EFHD2, EGR2, EMP1, EREG, ETV1, ETV4, ETV5, FAM43A, G0S2, GFPT2, HAS2, HES1, HIST1H1A, HIST1H2AK, HIST1H2BE, HIST1H2BF, HIST1H2BM, HIST1H3D, HIST1H3F, HIST1H4L, HIST2H3A, HMGA2, HPCAL1, IER3, ITGA2, KRT79, NT5E, OASL, PHLDA1, SHC3, SLC20A1, SLCO4A1, STC1, STX1A, TNFSF15*

Supplementary Table S4: Differentially expressed genes in RTK1 and RTK3 PPGL subclusters. Alphabetical list of genes significantly differentially expressed in RTK1 *vs* RTK3 clusters of PPGL in the Scandinavian (ANOVA $p < 0.05$, FDR < 0.25, fold change < -2 or > 2) and TCGA cohorts (student's T-test with Bonferroni correction factor 289 and 27 for under- and overexpressed genes, respectively); and mapped in MSigDB for gene set overlap analysis.

**RTK1 *vs* RTK3**	**Genes**
**Downregulated**	*ACTA2, ANGPTL2, AOC3, ASPN, BST2, C1R, C1S, CAV1, CAV2, CD53, CD68, CD86, CDH13, CFH, CNN1, COL15A1, COL1A2, COL3A1, COL4A1, COL4A2, COL6A3, CXCL10, CYR61, DCN, EDIL3, EFEMP1, EMP1, ENPEP, ENTPD1, EPB41L4A, EPS8, EVI2B, FAM26E, FAM69A, FCER1G, FCGR2A, FHL5, FLNA, FMO3, FPR3, FSTL1, FYB, GBP1, GBP2, GBP3, GIMAP4, GPR116, GPX8, HGF, IFI44L, IFITM1, IFITM3, IGHA1, IGHV3-33, IGHV3-74, IGJ, IGKC, IGKV1D-42, IGKV2D-30, IL1R1, IL2RG, ITGA2, ITGA5, ITGB5, JAG1, LAPTM5, LHFP, LTBP1, LUM, LY96, LYVE1, LYZ, MGP, MICAL2, MPZL2, MSR1, MYC, MYOF, NCKAP1L, NEXN, NFIB, OAS2, OLFML2B, OSMR, PDE1A, PDE4B, PDK4, PHACTR2, PITPNC1, PLN, PLS3, POSTN, PPIC, PRRX1, PRSS23, PTPRC, PXDN, SAMD9L, SERPINF1, SLFN11, SPARCL1, SRGN, ST6GAL1, SULF2, TAGLN, TES, TFPI, TGFBI, TGM2, THBS1, THBS2, TNC, TNFAIP3, TNFSF10, TNS1, TRIM22, VAMP8, VCAM1, VCAN, VSIG4, ZYX*
**Upregulated**	*PPP1R1B*

Supplementary Table S5: Primer pairs. Alphabetical list of forward (FP) and reverse (RP) primers used for RT-qPCR and Sanger sequencing (the latter marked with *). Primer sequences, amplicon lengths, and for RT-qPCR validated cDNA concentration ranges with *GUSB* (G) or *HPRT1* (H) as reference genes, are provided.

Gene	Sequence (5′→ 3′)	Amplicon length (bp)	Validated concentration range (ng/reaction)
ACTA2	FP:CGGGAGAAAATGACTCAAATTATGTT<br>RP: CATACATGGCTGGGACATTGAA	55	0.03-30 (G)
ANKRD1	FP: ACATACAAGACTCCTTCAGCCAACA<br>RP: TTTCCAGTGACCAGTTCCTCTACTTT	62	0.016-16 (H)
CCND1	FP: GAGGAGCTGCTGCAAATGG<br>RP: CGGCCAGGTTCCACTTGA	58	0.017-17 (G, H)
CITED2	FP: CCCGCCCTCGGTCTTC<br>RP: TTCCAGTCCTTCCGTTTTTGC	49	0.016-16 (H)
CMKLR1	FP: ATAACGGTGAATGAGAATGGAGGAT<br>RP: ACCGTAACTGATGGAAGTGTTGTAAT	55	0.016-16 (H)
GUSB	FP: CAAGACAGTGGGCTGGTGAATTA<br>RP: CTTGAACAGGTTACTGCCCTTGAC	57	n/A
HES1	FP:AGCACAGAAAGTCATCAAAGCCTATTAT<br>RP: ACTTTCATTTATTCTTGCTCTTCGTCTT	62	0.0075-7.5 (H)
HPRT1	FP: ATGGACTAATTATGGACAGGACTGAA<br>RP: CTCCCATCTCCTTCATCACATCT	60	n/A
HRAS	FP: CGGCAGGGAGTGGAGGAT<br>RP: GCTTGTGCTGCCGGATCT	58	0.03-30 (H)
NRAS*	FP: CAAACTGGTGGTGGTTGGAG<br>RP: AGTTCGTGGGCTTGTTTTGT	387	n/A
NRAS	FP: GACCAGACAGGGTGTTGAAGATG<br>RP: CATTCGGTACTGGCGTATTTCTCT	64	0.017-17 (H)
KIF1B*	FP: CGCACAGTGGTAGCAGTAGA<br>RP: CCATCTCATCTTGCTGCTCG	331	n/A
TAGLN	FP: CAAGGAGCTTTCCCCAGACAT<br>RP: GCACTTCGCGGCTCATG	62	0.015-15 (G)
TGFB2	FP: TTCTTCCCCTCCGAAACTGTCT<br>RP: ACTGAGCCAGAGGGTGTTGTAAC	53	0.016-16 (H)

Supplementary Table S6: PCR protocols. PCR protocol for amplicon in Sanger sequencing and RT-qPCR.

**Sanger sequencing**	**RT-qPCR**
**Start:** 1 minute, 95 °C<br>**35 cycles:**<br>Denaturation: 15 seconds, 95 °C<br>Annealing (*NRAS*) : 15 seconds, 57 °C<br>Annealing (*KIF1B*): 15 seconds, 50.5 °C<br>Extension: 45 seconds, 72 °C<br>End: 5 minutes, 72 °C	**Start:** 2 minutes, 50 °C<br>10 minutes, 95 °C<br>**40 cycles:**<br>Melting: 15 seconds, 95 °C<br>Annealing: 1 minute, 60 °C<br>**End:** Melt curve analysis
